# Global Expressions Landscape of NAC Transcription Factor Family and Their Responses to Abiotic Stresses in *Citrullus lanatus*

**DOI:** 10.1038/srep30574

**Published:** 2016-08-05

**Authors:** Xiaolong Lv, Shanrong Lan, Kateta Malangisha Guy, Jinghua Yang, Mingfang Zhang, Zhongyuan Hu

**Affiliations:** 1 Laboratory of Germplasm Innovation and Molecular Breeding, College of Agriculture and Biotechnology, Zhejiang University, Hangzhou, 310058, P. R. China; 2 Key Laboratory of Horticultural Plant Growth, Development & Quality Improvement, Ministry of Agriculture, Hangzhou, 310058, P. R. China; 3 Zhejiang Provincial Key Laboratory of Horticultural Plant Integrative Biology, Hangzhou, 310058, P. R. China

## Abstract

Watermelon (*Citrullus lanatus*) is one xerophyte that has relative higher tolerance to drought and salt stresses as well as more sensitivity to cold stress, compared with most model plants. These characteristics facilitate it a potential model crop for researches on salt, drought or cold tolerance. In this study, a genome-wide comprehensive analysis of the ClNAC transcription factor (TF) family was carried out for the first time, to investigate their transcriptional profiles and potential functions in response to these abiotic stresses. The expression profiling analysis reveals that several NAC TFs are highly responsive to abiotic stresses and development, for instance, subfamily IV NACs may play roles in maintaining water status under drought or salt conditions, as well as water and metabolites conduction and translocation toward fruit. In contrast, rapid and negative responses of most of the *ClNACs* to low-temperature adversity may be related to the sensitivity to cold stress. Crosstalks among these abiotic stresses and hormone (abscisic acid and jasmonic acid) pathways were also discussed based on the expression of *ClNAC* genes. Our results will provide useful insights for the functional mining of NAC family in watermelon, as well as into the mechanisms underlying abiotic tolerance in other cash crops.

The NAC [no apical meristem (NAM), *Arabidopsis thaliana* transcription activation factor (ATAF1/2) and cup-shaped cotyledon (CUC2)] gene family is one of the largest plant-specific transcription factor (TF) families. NAC proteins play key roles in regulating gene expression at the transcription level by binding to specific *cis*-acting elements in the promoters of target genes. Commonly, NAC proteins possess a conserved NAM domain at the N-terminus and a divergent transcription regulation domain at the C-terminus, forming the typical protein model of NAC transcription factors^1^,^2^. NAC domains are usually composed of nearly 150 amino acid residues and divided into five subdomains A–E^1^,^3^. Among them, subdomains C and D are conserved and bind to DNA. Subdomain A plays an important role in NAC dimeric proteins. Subdomains B and E are highly divergent and might confer functional diversity to NAC TFs^4^,^5^. The NAC domain’s crystal structure in *ANAC019* from *Arabidopsis* and in stress-response *NAC1* from rice were similar to the structure of WRKY^4^,^6^. Another study showed a high similarity between the protein domain structures of NAC and GLIA CELL MISSING (GCM)^7^. Therefore, NAC proteins are classified as members of the WRKY-GCM1 super family.

Increasing evidences indicate roles for NAC proteins in biological processes and transcriptional regulatory networks^8^. For example, ATAF1/2, CUC2, and ANAC036 are involved in cell division^9^,^10^,^11^. SECONDARY WALL NAC DOMAIN PROTEIN1 in rice and NAC SECONDARY WALL THICKENING PROMOTING FACTOR 2 (NST2) in *Arabidopsis* are concerned with the secondary growth^12^,^13^. CUC2 is involved in shoot apical meristem development^14^, AtNAM plays a role in embryo development^15^, ANAC029 (also known as AtNAP) and EPHEMERAL1 are involved in plant senescence^16^,^17^, AtNAC2 and TaNAC1 are implicated in lateral root development^18^,^19^, and some other NAC TFs play roles in nutrition transportation^20^, flowering time^21^, and cell death^22^. A tomato NAC gene is a positive regulator of carotenoid accumulation and fruit ripening^23^ and PpNAC1 activates the biosynthesis of anthocyanin in peach^24^, implying roles for NAC TFs in plant fruit development. Increasing amounts of evidence indicate that NAC is involved in xylem development^25^. The essential roles of the NAC family in both water-contributing and supporting cells indicated the contribution of this family to plants adaptation to land^26^.

NAC domain-containing proteins are also involved in plant abiotic and biotic responses. In *Arabidopsis*, *ANAC019*, *ANAC055* and *ANAC072* were markedly up-regulated by drought, salt, and abscisic acid (ABA) treatments, and consequently improve plant drought resistance^27^. Moreover, ANAC072 and ANAC019 also have the ability to positively regulate ABA signaling^27^,^28^,^29^. ANAC019 and ANAC055 can promote the expression of *VEGETATIVE STORAGE PROTEIN1* (*VSP1*) and *LIPOXYGENASE2* (*LOX2*), which are involved in jasmonic acid (JA) signaling^30^. In addition, the overexpression of a *Lepidium latifolium* NAC gene in tobacco enhanced its cold tolerance^31^. The *Ataf1-1* mutant showed decreased resistance to *Blumeria graminis f*.sp. hordei, suggesting a positive role for *ATAF1* in pathogen tolerance^32^, while *ATAF2* exhibited a positive response to JA and salicylic acid (SA)^33^.

*Citrullus lanatus* is one xerophyte that has relative higher tolerance to drought and salt stresses as well as more sensitivity to cold stress, compared with most other crops. However, a systematic analysis on ClNAC family genes and their responsive patterns to diverse abiotic stresses is lacking. Here, we identified 80 ClNAC TFs and predicted their induced patterns and functions through a genome-wide bioinformatics analysis. Furthermore, a global landscape of NAC expression patterns in response to abiotic stresses (drought, salt and cold) and phytohormones (ABA and JA) was investigated. This study will lay the basis of functional characterization of NAC TFs, as well as the advancement of research on abiotic tolerance in cash crops.

## Results and Discussion

### Identification of NAC TFs

**Figure 1:**
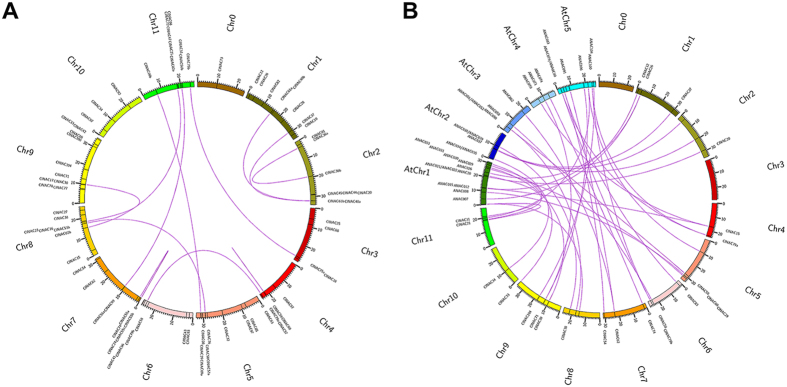
Visualization of the NAC TF linkage groups. **(A)** Chromosomal distributions of NAC TFs in the watermelon genome. The lines represent duplicate pairs of watermelon NAC genes. **(B)** Putative orthologs of NAC TFs in watermelon and *Arabidopsis*.

To identify ClNAC proteins, searches of the *Citrullus lanatus* genome using the BLASTp algorithm were performed with *Arabidopsis* and rice NAC proteins sequences as the query. In total, 80 putative NAC TFs with conserved NAM domain were identified (Table 1), which is in agreement with the watermelon NAC gene family in the Plant Transcription Factor Database (PlantTFDB; http://planttfdb.cbi.pku.edu.cn). The number of NAC TFs in watermelon is less (80) than in *Arabidopsis* (138) and rice (140). Owing to the lack of a designated standard annotation for the 80 NAC genes in watermelon, we named them *ClNAC1-ClNAC104* based on their homology to the *Arabidopsis* NAC proteins (highest to lowest sequence similarity level) and some numbers were omitted due to the lack of ANAC homologies in watermelon. The NAC TF genes identified in watermelon encoded proteins ranging from 153 to 642 amino acid (aa) residues in length, with an average of 346 aa (Table 1). Seventy-nine of the *ClNACs* were distributed across the 11 watermelon chromosomes, with *ClNAC73* putatively being located on the Chromosome 0 (Table 1, Fig. 1A). In an neighbor-joining (NJ) phylogenetic analysis, 12 pairs of duplicate/triplicate genes were identified, including two pairs of tandem duplicate genes (*ClNAC59* and *ClNAC60* on chromosome 4, and *ClNAC55b* and *ClNAC55c* on chromosome 7) (Fig. 1A; Supplementary Fig. S1). Most of the *ClNAC* duplicate genes had similar N-myristoylation motifs (Supplementary Fig. S2). These duplicate genes contributed significantly to the expansion of the watermelon NAC TF gene family. Simultaneously, 30 pairs of putative orthologs of NAC TFs, between watermelon and *Arabidopsis*, were found (Fig. 1B, Supplementary Figs S1 and S3).

### Phylogenetic analysis

**Figure 2:**
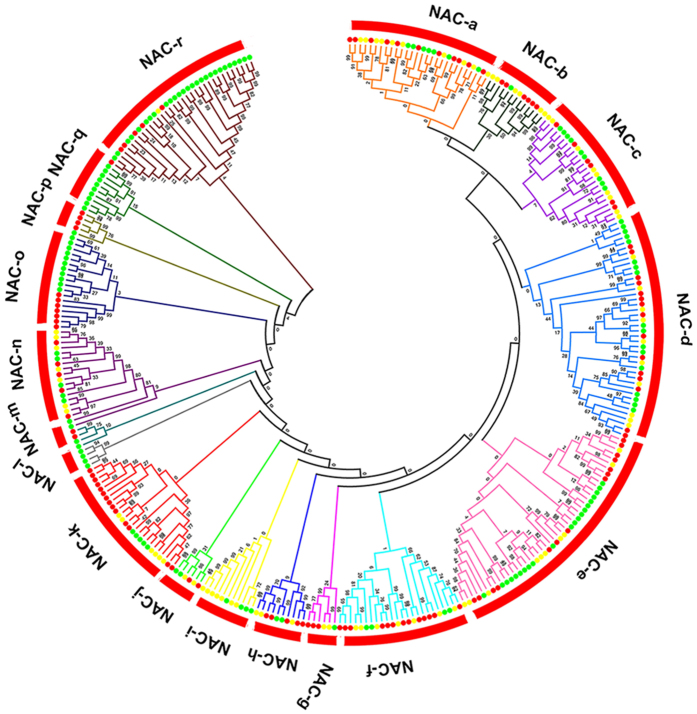
Phylogenetic tree of NAC proteins from watermelon, *Arabidopsis*, and rice. The phylogenetic tree is based on a sequence alignment of 329 NAC protein sequences from watermelon, *Arabidopsis*, and rice. The unrooted tree was generated with MEGA5.0 using the NJ method. Bootstrap values are indicated at each node. The NAC proteins are grouped into 18 distinct clades (a–r). The yellow, red, and green dots represent watermelon, *Arabidopsis*, and rice NACs, respectively.

To investigate the evolutionary relationships among the NAC TFs, 329 NAC domain sequences were predicted from *Arabidopsis*, rice, and watermelon using alignments of the full-length NAC sequences. These NAC proteins were classified into 18 groups (namely NAC-a to NAC-r; Fig. 2, Supplementary Fig. S1), which is in strong agreement with the results found in *Populus*^34^. NAC TFs in same group are likely to possess similar functions. For example, group NAC-a includes NAC proteins such as RD26, ANAC019, and ANAC055 and are involved in stress responses^28^,^30^, while group NAC-b possesses all of the NAC proteins, such as CUC1 and CUC2, that function in the delimitation of the shoot organ boundary^14^,^35^. The 80 ClNAC TFs are distributed throughout most of the groups, indicating multiple and various functions of NAC TFs in watermelon. Interestingly, ClNAC TF is absent in the NAC-m, NAC-o and NAC-p groups, which implies that these groups might be lost in watermelon during evolution. This finding may explain why watermelon contains fewer NAC TFs than *Arabidopsis*, even though these two plants have similar numbers of protein-coding genes. Similarly, group NAC-i did not contain any *Arabidopsis* members (Fig. 2, Supplementary Fig. S1). Additionally, group NAC-l and group NAC-q contain only rice members, suggesting that these groups were either acquired after the divergence of monocots and dicots, or were lost in watermelon and *Arabidopsis*.

### Gene structure and conserved motifs

**Figure 3:**
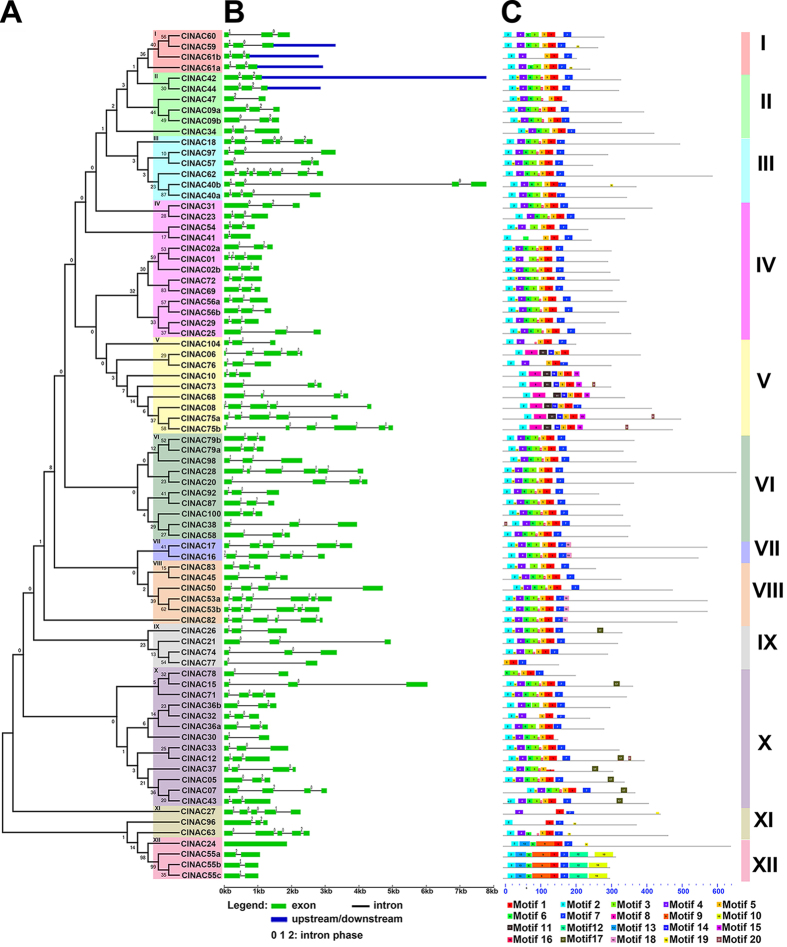
Phylogenetic relationships, gene structures and protein structures of the ClNAC TFs. **(A)** The phylogenetic tree was constructed with MEGA 5.0 using the NJ method with 1,000 bootstrap replicates based on a multiple alignment of 80 NAC amino acid sequences from watermelon. The 12 major subfamilies are indicated (I–XII) and are marked with different colored backgrounds. **(B)** Exon/intron structures of NAC genes from watermelon. Exons and introns are represented by green boxes and black lines, respectively. The sizes of the exons and introns are estimated using the scale at the bottom. **(C)** Schematic of the conserved motifs in the NAC proteins from watermelon elucidated by MEME. Every motif is represented by one colored box with a number. The black lines represent the non-conserved sequences. Refer to Supplementary Table S1 for individual motif details.

To get a better understanding of the structural diversity of ClNAC TFs, we compared the exon/intron organization in their coding sequences. The 80 ClNAC TFs were divided into 12 subfamilies in the NJ phylogenetic tree. Among them, subfamily IV and X with 13 members were the highest in numbers and subfamily VII was the lowest with only two members (Fig. 3A). Members in the same subfamily shared similar exon/intron structures in terms of intron phase, intron number, and exon length. For instance, the NAC genes in subfamily V and XI harbored two to four introns, while those in subfamily XII possessed only one intron, with the exception of *ClNAC24* which had no intron. By contrast, subfamily VII had the largest number of 4 to 5 introns. Interestingly, the intron number varied significantly, while the intron phase and exon length were highly conserved in subfamilies III, VI, and VIII (Fig. 3B).

To reveal the diversity of ClNAC TFs, the MEME program was used to predict putative motifs. Ultimately, 20 distinct motifs were identified (Supplementary Table S1). Most of the NAC TF proteins contained A to E motifs in the N-termini, which conferred DNA-binding activity^1^. Here, motif 2, 4, 3, 1 and 7 specified the NAM subdomains A to E, respectively. Most of the ClNAC proteins contain all of these five motifs, except for subfamily XII, which had no motif B, and subfamily V, which had neither subdomain A nor B. However, these two subfamilies had their specific motifs, such as motifs 9, 10, 12, and 13 in subfamily XII, and motifs 8, 11, 14, and 15 in subfamily V. Even if the divergence level in C-terminal regions of the NAC TF proteins was relatively high, some conserved motifs were also identified in these regions in some specific subfamilies, for example, motif 17 in subfamily X and motif 18 in subfamilies VII and VIII (Fig. 3C). These results suggested that the specific functions of different subfamilies might be owing to specific motifs.

### NAC gene response, localization and function predictions

**Figure 4:**
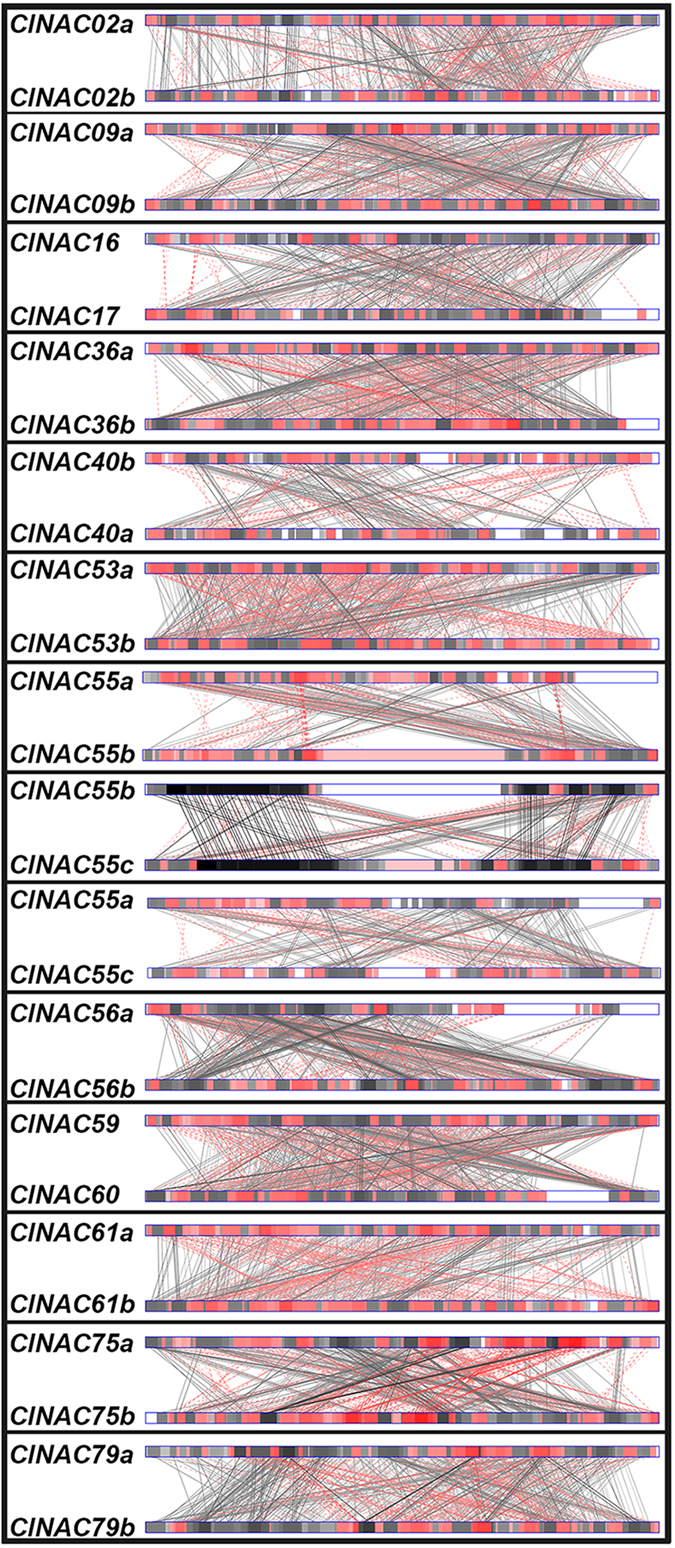
Comparative analysis of the promoter regions in ClNAC duplications. Black and red boxes with connecting lines between duplicate genes represent similar regions in their promoters. The depths of the different colors represent the similarities of conserved regions. Solid dark lines connect similar regions and red broken lines connect matched regions in the reversed orientation. White boxes without connecting lines represent divergent regions.

Gene expression responses are largely related to their promoters; therefore, we investigated the putative stimulus-responsive *cis*-elements in the promoter regions of all of the ClNAC genes (Supplementary Table S2). Nine types of *cis*-elements were detected, including *cis*-acting regulatory elements (AREs) that are essential for anaerobic induction; two *cis*-acting regulatory elements (TGACG-motif and CGTCA-motif) that are involved in MeJA responsiveness; MYB-binding sites (MBS) associated with drought inducibility; low-temperature-responsive elements (LTRs); ABA-responsive elements (ABREs); SA-responsive elements (TCA-elements); heat shock-responsive elements (HSEs) and ET-responsive elements (EREs)^36^,^37^,^38^,^39^,^40^,^41^. Every NAC gene contains at least one *cis*-element type in their promoter sequences (Supplementary Table S3), suggesting that these *ClNACs* are involved in watermelon response to different abiotic stresses and/or hormone signaling. Surprisingly, differences in the types and numbers of *cis*-elements were observed in some duplicate gene pairs. Two ERE elements exist in the promoter of *ClNAC09a*, while none could be found in its duplicate gene, *ClNAC09b* (Supplementary Table S3). A comparison of the promoter regions of all the duplicate gene pairs showed their divergence, although conserved regions were also observed (Fig. 4). Additionally, the protein’s function is related to its localization in some way^42^. Based on the subcellular localization predictions, most ClNACs probably function in the nucleus, while others were located in different organelles or the cytoplasm. For instance, ClNAC06, ClNAC62, ClNAC50, ClNAC53a and ClNAC74 might be located in chloroplasts; ClNAC07 and ClNAC30 might be located in mitochondria; and ClNAC01, ClNAC02b, ClNAC77, ClNAC78, and ClNAC104 might be located in the cytoplasm. Moreover, of the 80 ClNACs, only ClNAC06 contains a signal peptide, indicating that it has an important role in protein subcellular localization (Supplementary Fig. S4). Moreover, phosphorylation could adjust the cellular localization of TFs, and change their activities^43^. Each ClNAC protein sequence contains these three types of phosphorylation sites, with S phosphorylation being the most common (Supplementary Table S5, Supplementary Fig. S5). These phosphorylation sites might be involved in the regulation of protein activities when plants are subject to stresses.

### Expression profiles of *ClNACs* in tissues and fruit developmental stages

**Figure 5:**
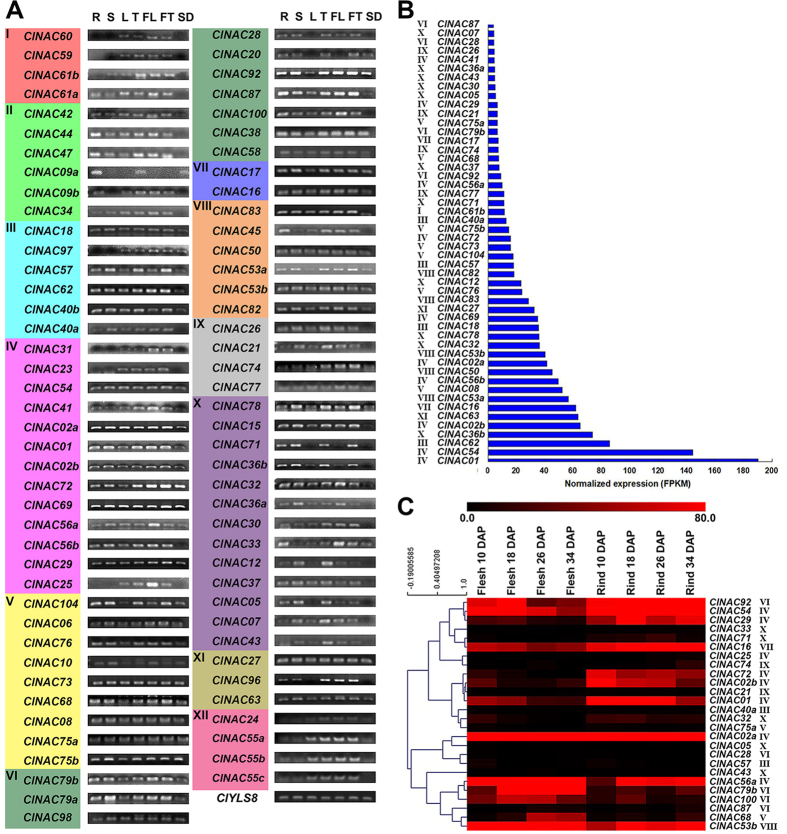
Expression profiles of watermelon NAC TFs in different tissues and at different fruit developmental stages. **(A)** RT-PCR analyses of NAC TFs in seven watermelon tissues. R: root; S: stem apex; L: young leaf; T: tendril; FL: flower; F: young fruit; SD: seed. *CIYLS8* was used as the control. **(B)** The transcript levels of NAC TFs in watermelon vascular tissues. Numbers on the vertical axis represent the normalized expression (FPKM) of 50 NAC TFs in watermelon vascular tissues. **(C)** Expression profiles of watermelon NAC TFs across different fruit developmental stages. The scale representing the relative signal intensity values is shown above. DAP: Days After Pollination.

In total, 45 NAC TFs could be detected in all of the tissues, suggesting that they may have various regulatory roles in multiple tissues at multiple developmental stages. Besides, the expression of all *ClNACs* can be detected in young fruit, except for *ClNAC09a*. While subfamily XII exhibit the most uniform expression pattern, and all of the members could be detected in tissues of young leaf, tendril, flower, and young fruit. Furthermore, most duplicated gene pairs shared similar expression patterns (Fig. 5A).

As a drought-tolerant crop with a high water demand, a powerful vascular system is essential for watermelon to maintain its water status to keep homeostasis under water-deficit conditions. Moreover, increasing evidence indicates that NAC TFs play important roles in the development of vascular tissues^2^,^25^,^44^,^46^, as well as in the adaptation of plants to land^26^. To determine the functions of ClNAC TFs in the development of vascular system, we analyzed the normalized expression of ClNAC TFs using published transcriptome sequencing data^45^. *ClNAC54* and *ClNAC01*, which belong to the subfamily IV, show extremely higher expression levels. The expression levels of six *ClNAC* genes (*ClNAC07*, *ClNAC05*, *ClNAC26*, *ClNAC30*, *ClNAC24*, and *ClNAC37*), homologous to *Arabidopsis VASCULAR-RELATED NAC-DOMAIN* (*VND*) genes^25^,^44^,^46^, were relatively lower. The expression of *ClNAC43* and *ClNAC18* were also detected, and their putative homologs, *NST1* and *SECONDARY WALL-ASSOCIATED NAC DOMAIN 1* (*SND1*), play crucial roles in secondary wall thickening^47^,^48^. Interestingly, 10 out of 13 subfamily IV members had detectable expression levels that were mostly relatively higher (Fig. 5B), indicating that subfamily IV may be involved in the vascular system development. The occurrence of the plant vascular system is a striking innovation that enabled its colonization of land, and NAC proteins played essential roles in the adaptation of plants to land^26^. The putative functions of subfamily IV ClNACs in vascular development suggested that subfamily IV is likely involved in the evolutionary process of water conduction in watermelon.

Given the expression of almost all *ClNACs* in young fruit (Fig. 5A), we analyzed the involvement of NAC TFs in different parts of the fruit during different fruit stages (Fig. 5C). The expression levels of *ClNAC16*, *ClNAC92*, *ClNAC54*, and *ClNAC29* were relatively higher in the rind at all of the stages, while their expression in the flesh was higher in the early stages and decreased from 26 days after pollination. Moreover, the transcript levels of *ClNAC32*, *ClNAC72*, *ClNAC02b*, and *ClNAC01* were higher in the rind than in the flesh, and their expression levels were relatively higher in the earlier stages of each tissue development, which suggested that these genes might play more important roles in the early stages of rind development. However, some *ClNACs*, such as *ClNAC56a*, *ClNAC79b*, *ClNAC100*, and *ClNAC53b*, showed relatively higher levels in the later stages (Fig. 5C). These results indicated that different NAC TFs play roles in different fruit ripening stages. Most (10 of 12) of the highly expressed genes detected in this analysis belonged to subfamily IV or VI, indicating that these two subfamilies might be important for fruit development. The vascular system is essential for water and sugar transportation during fruit development. Here, 21 common *ClNACs* were detected in both vascular tissues and fruit, with 10 of them belonging to subfamily IV or VI (Fig. 5B,C). This suggested that these two subfamilies were important in correlating the development of vascular tissues and fruit in watermelon. In particular, *ClNAC01*, *ClNAC02a* and *ClNAC02b*, which presented quite high expression levels in both vascular and fruit (Fig. 5B,C), were similar to *SlNAC4* in protein sequence and expression profiles. This tomato NAC gene is a positive regulator of carotenoid accumulation and fruit ripening^23^. Additionally, TtNAM-B1, which had a sequence similarity with ClNAC56a and ClNAC56b, increases nutrient remobilization in wheat^20^. All of these *ClNACs* belong to subfamily IV, implying that this subfamily is important for the transport of nutrients and metabolites to watermelon fruit via the vascular system.

### Expression profiles of the *ClNACs* under abiotic stress

**Figure 6:**
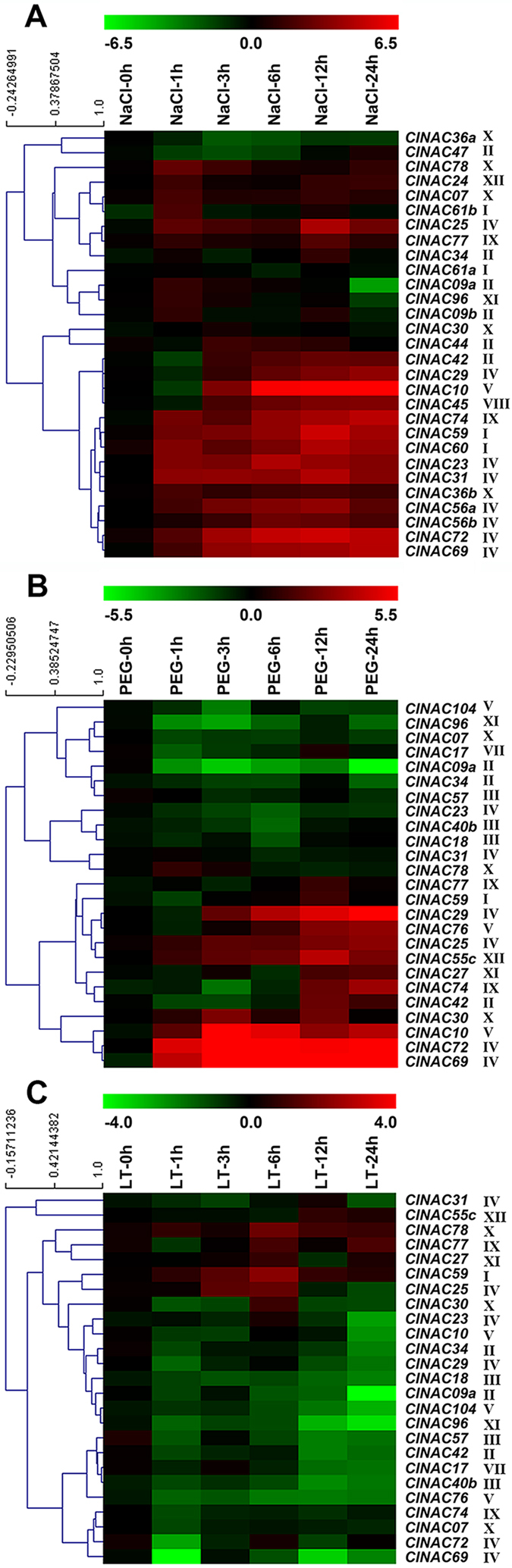
Expression analyses of NAC TFs in the roots of watermelon exposed to NaCl, PEG and low temperature. Expression analysis of NAC TFs in the roots of watermelon exposed to 200 mM NaCl. **(B)** Expression analysis of 23 NAC TFs in the roots of watermelon exposed to 20% PEG. **(C)** Expression analysis of 23 NAC TFs in the roots of watermelon exposed to 8 °C (low-temperature, LT). The scale representing the relative signal intensity values is shown above. Hierarchical clustering was used in the data analysis.

Given that *Citrullus lanatus* is tolerant to salt and drought stresses, but sensitive to low temperatures; and NAC TFs are likely to be involved in physiological adaptations in response to these stresses^18^,^49^,^50^. We examined the expression levels of some *ClNACs* under salt, drought and low-temperature treatments. Salt stress caused quick and significant responses of 10 *ClNACs* (*ClNAC74*, *ClNAC59*, *ClNAC60*, *ClNAC23*, *ClNAC31*, *ClNAC36b*, *ClNAC56a*, *ClNAC56b*, *ClNAC72*, and *ClNAC69*) in roots. It also caused a quick but transient increase in the expression level of nine *ClNACs* (*ClNAC78*, *ClNAC24*, *ClNAC07*, *ClNAC61b*, *ClNAC25*, *ClNAC77*, *ClNAC09a*, *ClNAC96*, and *ClNAC09b*) (Fig. 6A). In *Arabidopsis*, there are three closely related *stress-response* NAC genes (*ANAC019*, *ANAC055* and *ANAC072*), which were induced by drought, salinity, and the hormones ABA and JA^27^,^28^,^29^,^30^. Here, their watermelon orthologs (*ClNAC72* and *ClNAC69*) also showed positive responses to the NaCl treatment. Notably, all of these members of subfamily IV were extremely sensitive to NaCl treatment, which is in strong agreement with their functions in vascular development. Furthermore, 3 quarters members of subfamily I also showed rapid and positive responses after the NaCl treatment. The high response of subfamilies I and IV NACs to salt treatment provided primary evidence for their possible participation in plant salt stress tolerance.

After PEG treatment, several genes, including *ClNAC29*, *ClNAC25*, *ClNAC55c*, *ClNAC30*, *ClNAC10*, *ClNAC72* and *ClNAC69*, showed rapid and positive responses (Fig. 6B). Among them, *ClNAC72* and *ClNAC69* were the most outstanding responsers. In contrast, there were about half of the detected *ClNAC* genes were quickly and markedly down-regulated. Among them, *ClNAC96* and *ClNAC09a* showed the most significant decrease, suggesting their potential involvement in drought tolerance in a negative manner (Fig. 6B). Interestingly, four highly expressed *ClNACs* (*ClNAC29*, *ClNAC25*, *ClNAC72* and *ClNAC69*) belonged to subfamily IV, which also participates in vascular development and salt response (Figs 5B and 6A). As the function of *ANAC019*, *ANAC055* and *ANAC072*, homologs of *ClNAC72* and *ClNAC69*, in drought tolerance have been demonstrated in transgenic plant^27^, and proteins with similar structure have the same kinds of function, we hypothesized that subfamily IV ClNACs may play similar roles for plant responses to water stresses.

Under low-temperature stress, most of the detected *ClNACs* showed negative responses (Fig. 6C), which was assumed to be attributed to the sensitivity of watermelon to this stress. There were also few *ClNAC* genes that were induced by the low-temperature. Among them, *ClNAC25*, *ClNAC78* and *ClNAC59*, exhibited quicker responses to the low-temperature and higher fold changes in expression levels than the others. Almost all of positive-responding genes showed their expressional peak at 6h after treatment, suggesting their earlier responses to low-temperature stress (Fig. 6C). Additionally, the LTR element, which is responsible for low-temperature inducibility, could only be found in the promoters of some *ClNAC* genes, such as *ClNAC30*, *ClNAC31*, *ClNAC55c* and *ClNAC77* (Supplementary Table S3), and all of these *ClNACs* were found to be up-regulated under low temperature. Notably, there were four *ClNACs* (*ClNAC25*, *ClNAC77*, *ClNAC78* and *ClNAC59*), exhibited positive response to drought, salt and low-temperature stresses, implying their involvement in the crosstalk of abiotic stress signal pathways.

### Expression profiles of the *ClNACs* in response to exogenous ABA and JA

**Figure 7:**
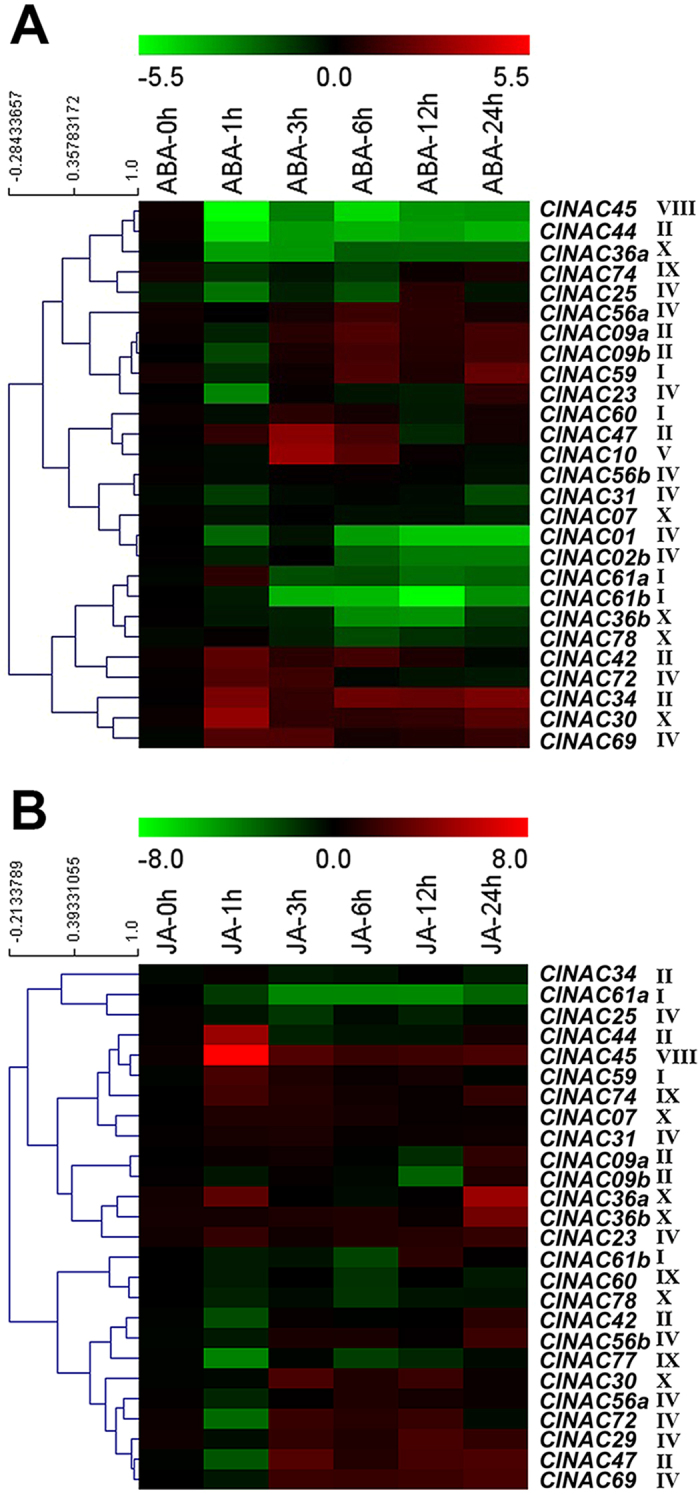
Expression analyses of NAC TFs in the roots of watermelon exposed to ABA and JA. **(A)** Expression analysis of NAC TFs in the roots of watermelon exposed to ABA. **(B)** Expression analysis of 24 NAC TFs in the roots of watermelon exposed to JA. The scale representing the relative signal intensity values is shown above. Hierarchical clustering was used in the data analysis.

Given that ABA plays crucial roles in response to environmental stresses^51^,^52^,^53^, the response of several selected NAC TFs to exogenous ABA were examined (Fig. 7A). There are five *ClNACs* (*ClNAC42*, *ClNAC72*, *ClNAC34*, *ClNAC30* and *ClNAC69*) that showed positive responses quickly and persistently. Whereas, the expression of some *ClNACs* (*ClNAC74*, *ClNAC25*, *ClNAC56a*, *ClNAC09a*, *ClNAC09b*, *ClNAC59*, *ClNAC23*, and *ClNAC60*) was significantly enhanced after a transient inhibition. Not surprisingly, ABRE elements were observed in most of their promoters (Fig. 7A, Supplementary Table S3). In contrast, some negative responding *ClNACs* (*ClNAC45*, *ClNAC44*, *ClNAC36a*, *ClNAC01*, *ClNAC02b*, *ClNAC61b*, *ClNAC36b*, and *ClNAC78*) were also found. Interestingly, *ClNAC56a*, *ClNAC59*, and *ClNAC60* positively responded to both NaCl and ABA treatments, and *ClNAC72*, *ClNAC69*, *ClNAC42* and *ClNAC10* were up-regulated by PEG, NaCl and ABA treatments. Moreover, *ClNAC25* was induced by salt, drought, low-temperature, and ABA treatments (Figs 6 and 7A). Thus, these *ClNACs* might confer abiotic stress responses through the ABA pathway. Additionally, there are some *ClNAC* genes, such as *ClNAC07*, *ClNAC56b*, *ClNAC31*, *ClNAC36b* and *ClNAC74*, that were highly up-regulated by abiotic stress, but not enhanced by ABA treatment (Figs 6A,B and 7A), implying that they may participate in responses to abiotic stresses via an ABA-independent pathway.

JA is an important hormone that regulates plant defense responses against biotic stresses, as well as a moderator of abiotic tolerance^54^,^55^. Thus, we analyzed the expression of *ClNACs* in response to JA. Some *ClNACs* showed positive responses to the exogenous JA treatment, which might result from the MeJA-responsiveness *cis*-acting regulatory elements (T GACG-motif and CGTCA-motif) present in most of the *ClNAC* promoters (Fig. 7B; Supplementary Table S3). Several *ClNACs* (*ClNAC29*, *ClNAC23*, *ClNAC31*, *ClNAC56b*, *ClNAC44*, *ClNAC45*, *ClNAC36b*, *ClNAC72*, *ClNAC69*, and *ClNAC74*) positively responded to both NaCl and JA treatments (Figs 6A and 7B), implying that they participate in salt stress responding via the JA pathway. Interestingly, *ClNAC77* and *ClNAC78* may participate in responding to all abiotic stresses above through ABA- and JA-independent pathways, as they showed no or negative responses to ABA or JA. While some other *ClNACs* (*ClNAC59*, *ClNAC47*, *ClNAC30*, *ClNAC72* and *ClNAC69*) were induced by both JA and ABA treatments (Fig. 7A,B), suggesting that they may be the common targets downstream of the ABA- and JA-mediated stress responses. In *Arabidopsis*, *ANAC072* and *ANAC019* have the ability to positively regulate ABA signaling^28^,^29^. Moreover, *ANAC019* and *ANAC055* function as activators of JA-signaled defense responses^30^. Here, *ClNAC72* and *ClNAC69* exhibited similar expression patterns as those of their putative homologs (*ANAC072*, *ANAC019* and *ANAC055*) under NaCl, PEG, ABA and JA treatments (Fig. 7)^27^. This suggests that *ClNAC72* and *ClNAC69* may also act as positive regulators of ABA and JA signaling in salt and drought responses. Notably, all of the subfamily IV NACs, involved in abiotic stress responses, were mediated by ABA and/or JA treatment (Figs 6 and 7). This implies that subfamily IV may be important downstream regulators of ABA- and/or JA- signal-induced stress defenses.

In conclusion, we selected 80 NAC genes and classified them into subfamilies based on their amino acid sequences for the first time in watermelon. Here we showed a global expression landscape of NAC TFs in response to various abiotic stresses. The watermelon ClNACs from different subfamilies exhibited diverse responsive patterns to environmental adversity. However, some subfamilies are highly responsive to abiotic stresses, such as salinity, cold and water deficiency, as well as involved in some distinctive vascular tissue and fruit development. The results also uncovered that the sensitivity of watermelon to cold stress might be related to the rapid and negative response of NAC TFs to low-temperature exposure. Given further studies are still needed to unravel the roles of ClNACs in the regulation of plant abiotic tolerance, our findings provide valuable clues for further functional research on NAC TF family in crop and its adaptation improvement to abiotic stresses via molecular approaches.

## Methods

### Plant materials, growth conditions and stress treatments

Watermelon of *Citrullus lanatus* cv. IVSM9 seedlings were used in this study. For the abiotic stress conditions, watermelon seedlings three true-leaves stage were grown in Hoagland solution containing 200 mM NaCl, 20% PEG6000 (w/v), 100 μΜ ABA, and 50 μΜ JA, respectively, under a photoperiod of 16 h at 27 °C (day) and 8 h at 24 °C (night) in a phytotron. The low-temperature treatment was carried out at 8 °C under the same photoperiod.

### Sequence database searches

To identify the watermelon NAC TF gene family, *Arabidopsis* (https://www.arabidopsis.org/) and rice (http://rapdb.dna.affrc.go.jp) NAC TF protein sequences were used to search the watermelon genome database (version 1; http://www.icugi.org/) using BLASTP, and then, a self-BLAST of the sequences was performed to remove redundancy. All of the putative candidates were manually verified using NCBI (http://www.ncbi.nlm.nih.gov/) to confirm the presence of the protein NAM conserved domain. They were then further examined to obtain all of the protein sequences using SMART (http://smart.embl-heidelberg.de/) and Pfam (http://pfam.sanger.ac.uk). Finally, all of the obtained protein sequences were compared with the watermelon NAC TF sequences downloaded from the PlantTFDB (http://planttfdb.cbi.pku.edu.cn/).

### Phylogenetic analysis

Multiple sequence alignments of the full-length amino acid sequences were aligned using Clustal W. The unrooted phylogenetic trees were constructed according to the NJ method using MEGA 5.0, and the bootstrap test was carried out with 1,000 iterations.

### Gene homologs and chromosomal location

The duplicate genes and the homologous genes between watermelon and *Arabidopsis*, based on the NAC protein phylogenetic tree from watermelon, *Arabidopsis*, and rice, were identified using the protocol of Kong *et al.*^56^. The tandem duplicated genes were identified and are defined as an array of two or more genes that were in the same phylogenetic group and found within a 100-kb chromosomal fragment^57^. All of the NAC genes chromosomal locations were found in the Cucurbit Genomics Database and then were visualized in a Circos map using CIRCOS software (http://circos.ca).

### Genomic structure and conserved motifs

The Gene Structure Display Server (GSDS; http://gsds.cbi.pku.edu.cn/) program was used to elucidate the exon/intron organization of NAC genes. The Multiple Expectation Maximization for Motif Elicitation (MEME; http://meme-suite.org/) program was used to illustrate the motifs in 80 putative ClNAC protein sequences.

### Prediction of promoter *cis*-elements, subcellular localizations, phosphorylation sites, and signal peptides

The putative *cis*-acting regulatory DNA elements (*cis*-elements) in the promoter regions of NAC genes were identified using the PlantCARE (http://bioinformatics.psb.ugent. be/webtools/plantcare/html/) program. *Cis*-elements were identified within the 1000-bp genomic DNA sequence upstream of the initiation codon (ATG)^58^. The GATA program was used to perform a comparative analysis of the promoter regions^59^. WoLF PSORT (http://wolfpsort.seq.cbrc.jp) was used to predict the subcellular localization, while phosphorylation sites and signal peptides were identified using NetPhos2.0 Server (http://www.cbs.dtu.dk/services/NetPhos/) and SignalP (http://www.cbs.dtu.dk/services/SignalP), respectively.

### Expression patterns analyses by RT-PCR and qRT-PCR

Total RNA was extracted from all of the tissue samples using Trizol reagent (Invitrogen, Carlsbad, CA, USA) according to the manufacturer’s instructions. First-strand cDNAs were synthesized using the Transcriptor First Strand cDNA Synthesis kit (Roche, Switzerland). To detect PCR products, 2% agarose gel electrophoresis was used. qRT-PCR reactions were performed in the ABI PRISM 7900HT (Applied Biosystems, USA) using FastStart Universal SYBR Green Master (Roche, Switzerland) according to the manufacturer’s instructions. The relative expression levels of NAC genes were calculated according to the method of Livak and Schmittgen^60^. The primers used in this analysis are described in Supplementary Table S6.

### Transcriptome sequencing data analysis

The transcriptome sequencing data for vascular and fruit developmental stages were obtained from a published paper^45^ using the identified ClNAC ID. The expression profiles were analyzed and visualized by MeV4.9.0 software (The Institute for Genomic Research, USA).

## Additional Information

**How to cite this article**: Lv, X. *et al.* Global Expressions Landscape of NAC Transcription Factor Family and Their Responses to Abiotic Stresses in *Citrullus lanatus. Sci. Rep.* **6**, 30574; doi: 10.1038/srep30574 (2016).

## Supplementary Material

Supplementary Information

Supplementary Dataset

## Figures and Tables

**Table 1:** NAC transcription factor gene family in watermelon.

Gene symbol	Gene locus	Length(aa)	Gene Location	Putative *Arabidopsis* orthologs	The closest genes	E-value
*ClNAC01*	Cla007853	289	Chr2:1955638..1956791		*ANAC002/ATAF1*	1.00E-103
*ClNAC02a*	Cla023182	299	Chr11:18231295..18232770	*ANAC002/ATAF1*		1.00E-135
*ClNAC02b*	Cla013922	296	Chr8:14908050..14909118	*ANAC002/ATAF1*		1.00E-140
*ClNAC05*	Cla006268	334	Chr5:7417554..7418938		*ANAC007/VND4*	1.00E-114
*ClNAC06*	Cla004626	379	Chr9:31582624..31584970	*ANAC008*		5.00E-76
*ClNAC07*	Cla005677	363	Chr10:3535524..3538561		*ANAC007/VND4*	1.00E-105
*ClNAC08*	Cla020366	410	Chr5:30465691..30470090		*ANAC008*	1.00E-142
*ClNAC09a*	Cla010181	388	Chr5:31397122..31398806		*ANAC009*	1.00E-104
*ClNAC09b*	Cla003347	327	Chr11:7128468..7130130		*ANAC009*	4.00E-82
*ClNAC10*	Cla009648	212	Chr1:31813698..31814520		*ANAC010/SND3*	2.00E-79
*ClNAC12*	Cla011325	389	Chr1:1270767..1272154	*ANAC012*/*SND1*/*NST3*		1.00E-107
*ClNAC15*	Cla012377	358	Chr8:2676356..2682444	*ANAC070, ANAC015*		E-112, 2E-90
*ClNAC16*	Cla013643	538	Chr8:18071416..18074448		*ANAC016*	1.00E-126
*ClNAC17*	Cla016331	562	Chr9:9847279..9851129		*ANAC017*	1.00E-125
*ClNAC18*	Cla011315	487	Chr3:27577243..27579904		*ANAC018/NTL9*	1.00E-52
*ClNAC20*	Cla013445	361	Chr2:29297528..29301836	*ANAC20*		1.00E-102
*ClNAC21*	Cla023219	317	Chr11:18613972..18618963	*ANAC021/ANAC022/AtNAC1*		1.00E-108
*ClNAC23*	Cla021917	336	Chr8:18558531..18559867		*ANAC031/CUC3*	1.00E-76
*ClNAC24*	Cla002713	625	Chr7:279898..281775		*ANAC030/VND7*	5.00E-14
*ClNAC25*	Cla019475	353	Chr3:5597680..5600578		*ANAC025*	4.00E-93
*ClNAC26*	Cla011554	329	Chr1:3745458..3747358	*ANAC007/VND4, ANAC026*		E-112, E-104
*ClNAC27*	Cla022514	433	Chr8:24287182..24289453		*ANAC028*	9.00E-09
*ClNAC28*	Cla009127	642	Chr1:22943282..22947465		*ANAC028*	1.00E-152
*ClNAC29*	Cla010201	283	Chr5:31276248..31277300	*ANAC029/ATNAP/NAP*		1.00E-111
*ClNAC30*	Cla016349	153	Chr9:9685786..9687158	*ANAC030/VND7*		2.00E-88
*ClNAC31*	Cla023471	411	Chr11:20978854..20981134	*ANAC031/CUC3*		4.00E-98
*ClNAC32*	Cla002170	240	Chr5:20029237..20030302		*ANAC083*	2.00E-40
*ClNAC33*	Cla005472	320	Chr9:34952827..34954767	*ANAC033*		1.00E-103
*ClNAC34*	Cla004555	416	Chr10:10290284..10291953	*ANAC034/ANAC035*		1.00E-110
*ClNAC36a*	Cla015772	279	Chr2:3373610..3374942	*ANAC036*		1.00E-109
*ClNAC36b*	Cla006906	296	Chr2:19551544..19553134	*ANAC036*		1.00E-97
*ClNAC37*	Cla014269	303	Chr1:29585085..29587217	*ANAC037/VND1*		6.00E-97
*ClNAC38*	Cla022231	351	Chr8:21813445..21817442	*ANAC038/ANAC039*		1.00E-108
*ClNAC40a*	Cla008629	341	Chr2:32424448..32427343		*ANAC040/NTL8*	3.00E-84
*ClNAC40b*	Cla011058	367	Chr1:16131062..16138877		*ANAC040/NTL8*	7.00E-89
*ClNAC41*	Cla019304	244	Chr6:26797719..26798537		*ANAC083*	1.00E-29
*ClNAC42*	Cla005508	325	Chr9:34507930..34509090		*ANAC042*	4.00E-81
*ClNAC43*	Cla006697	400	Chr6:3348856..3350249		*ANAC043/NST1, ANAC066*	8E-94, 5E-80
*ClNAC44*	Cla013474	319	Chr2:28979835..28981165		*ANAC042*	3.00E-81
*ClNAC45*	Cla013475	326	Chr2:28970371..28972299		*ANAC042*	9.00E-78
*ClNAC47*	Cla023239	176	Chr11:18821525..18822788		*ANAC042*	6.00E-76
*ClNAC50*	Cla020528	467	Chr5:29010431..29015195	*ANAC050, ANAC051/ANAC052*		3E-93, 5E-91
*ClNAC53a*	Cla020527	563	Chr5:29018616..29021862	*ANAC053*, *NAC2*		1E-145，1E-117
*ClNAC53b*	Cla013731	563	Chr8:17220446..17223318	*ANAC053*, *NAC2*		1E-134，1E-111
*ClNAC54*	Cla010881	235	Chr7:30741029..30741978		*ANAC083*	2.00E-95
*ClNAC55a*	Cla002217	310	Chr7:786430..787518		*ANAC056/AtNAC2*	1.00E-09
*ClNAC55b*	Cla002680	294	Chr7:5608..6648		*ANAC056/AtNAC2*	6.00E-10
*ClNAC55c*	Cla002681	294	Chr7:10329..11369		*ANAC056/AtNAC2*	6.00E-10
*ClNAC56a*	Cla011760	340	Chr7:10715001..10716327		*ANAC056/AtNAC2*	1.00E-108
*ClNAC56b*	Cla023408	320	Chr11:20484013..20485439		*ANAC056/AtNAC2*	1.00E-101
*ClNAC57*	Cla018634	248	Chr4:23877496..23880341		*ANAC057*	1.00E-136
*ClNAC58*	Cla018973	345	Chr6:23992729..23994725	*ANAC058*		2.00E-91
*ClNAC59*	Cla018410	262	Chr4:21728169..21729673		*ANAC090*	3.00E-69
*ClNAC60*	Cla018411	279	Chr4:21736304..21738286		*ANAC090*	1.00E-72
*ClNAC61a*	Cla003039	240	Chr1:15383814..15384842	*ANAC090, ANAC061*		5E-63, 4E-61
*ClNAC61b*	Cla008633	203	Chr2:32391575..32392368	*ANAC090, ANAC061*		8E-64, 4E-60
*ClNAC62*	Cla002400	576	Chr7:23188084..23191054	*ANAC091, ANAC062*		4E-83,4E-82
*ClNAC63*	Cla021063	448	Chr5:85305..87840		*ANAC062*	1.00E-30
*ClNAC68*	Cla019693	336	Chr3:8593061..8596774		*ANAC073*	1.00E-101
*ClNAC69*	Cla011761	302	Chr7:10570728..10571833		*ANAC072/RD26*	1.00E-115
*ClNAC71*	Cla016169	341	Chr9:12586253..12587805	*ANAC096, ANAC071*		2E-87, 2E-82
*ClNAC72*	Cla023407	321	Chr11:20462289..20463440		*ANAC072/RD26*	1.00E-125
*ClNAC73*	Cla000378	298	Chr0:10012381..10015299		*ANAC073*	1.00E-112
*ClNAC74*	Cla005970	289	Chr7:1814933..1818320	*ANAC074*		2.00E-78
*ClNAC75a*	Cla011248	490	Chr3:26766834..26770238		*ANAC075*	1.00E-138
*ClNAC75b*	Cla016810	467	Chr11:25081349..25086384		*ANAC075*	1.00E-139
*ClNAC76*	Cla020655	299	Chr5:27964577..27965999		*ANAC074*	2.00E-14
*ClNAC77*	Cla014880	154	Chr9:7121234..7124037		*ANAC074*	5.00E-29
*ClNAC78*	Cla014910	201	Chr9:7520978..7522919		*ANAC074*	7.00E-55
*ClNAC79a*	Cla018596	332	Chr4:23607586..23608786	*ANAC100/ATNAC5*		E-103, 3E-97
*ClNAC79b*	Cla019099	362	Chr6:25092288..25093541	*ANAC100/ATNAC5*		E-115, E-106
*ClNAC82*	Cla008434	480	Chr1:9372652..9375621		*ANAC082, ANAC103*	2E-67, 2E-67
*ClNAC83*	Cla001495	256	Chr6:1853182..1854287	*ANAC083*		1.00E-107
*ClNAC87*	Cla012144	323	Chr4:15660560..15662087		*ANAC087, ANAC046*	3E-78, 7E-73
*ClNAC92*	Cla016990	265	Chr10:21307912..21309583		*ANAC092/ATNAC2/ATNAC6*	7.00E-81
*ClNAC96*	Cla019229	367	Chr6:26229431..26230741		*ANAC096*	2.00E-13
*ClNAC97*	Cla004290	289	Chr5:9565473..9568819	*ANAC098/CUC2*		8.00E-93
*ClNAC98*	Cla023357	368	Chr11:19954210..19956573	*ANAC098/CUC2*		1.00E-107
*ClNAC100*	Cla010317	331	Chr9:30221670..30222842		*ANAC100/ATNAC5*	2.00E-96
*ClNAC104*	Cla009439	202	Chr9:17570927..17572481	*ANAC104/XND1*		5.00E-59
