# Supplementary material for: Global Expressions Landscape of NAC Transcription Factor Family and Their Responses to Abiotic Stresses in Citrullus lanatus
Source: Sci Rep. 2016 Aug 5;6:30574. doi: 10.1038/srep30574 (PMC4974498; doi:10.1038/srep30574)
Supplement: Supplementary Information [file srep30574-s1.pdf]

Global Expressions Landscape of NAC Transcription Factor Family and Their Responses to Abiotic Stresses in *Citrullus lanatus*

Xiaolong Lv[1], Shanrong Lan[1], Kateta Malangisha Guy[1], Jinghua Yang[1,2,3], Mingfang Zhang[1,2,3], Zhongyuan Hu[1,2,3,*]

[1] Laboratory of Germplasm Innovation and Molecular Breeding, College of Agriculture and Biotechnology, Zhejiang University, Hangzhou, 310058, P.R.China

[2] Key laboratory of Horticultural Plant Growth, Development & Quality Improvement, Ministry of Agriculture, Hangzhou, 310058, P.R.China

[3] Zhejiang Provincial Key Laboratory of Horticultural Plant Integrative Biology, Hangzhou, 310058, P.R.China

[*] Corresponding author: Zhongyuan Hu

Supplemental Figures and Tables

Figure S1. Phylogenetic tree of NAC proteins from watermelon, *Arabidopsis* and rice.

Figure S2. Protein structures of 12 pairs of duplicate genes from watermelon.

Figure S3. Protein structures of 30 pairs of putative orthologs of NAC TFs between watermelon and *Arabidopsis*.

Figure S4. Signal peptides predictions in 80 ClNACs.

Figure S5. Phosphorylation site predictions in 80 ClNACs.

Table S1. Sequence logos for the conserved motifs of watermelon NAC domain proteins.

Table S3. Number of different cis-elements presented in the promoter regions of the *ClNAC* genes.

Table S4. Subcellular localization predictions for ClNACs

Table S5. Phosphorylation site predictions in 80 ClNACs.

Table S6. Primer sequences of *ClNAC* genes for RT-PCR and qRT-PCR analyses.

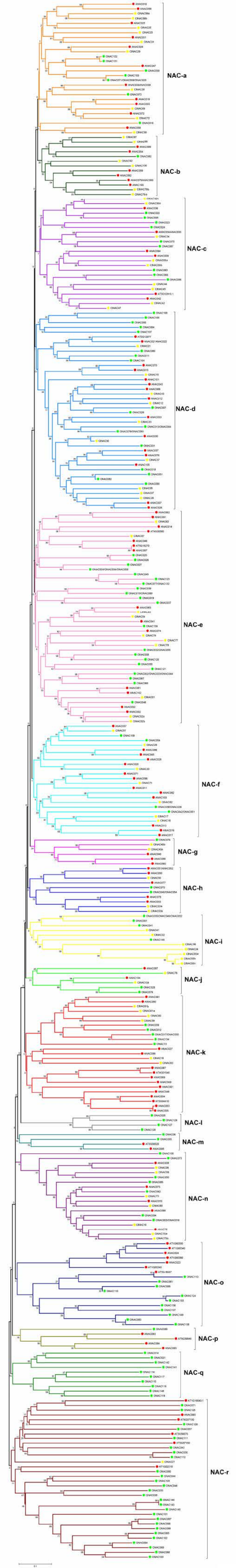
NAC-a
NAC-b
NAC-c
NAC-d
NAC-e
NAC-f
NAC-g
NAC-h
NAC-i
NAC-j
NAC-k
NAC-l
NAC-m
NAC-n
NAC-o
NAC-p
NAC-q
NAC-r

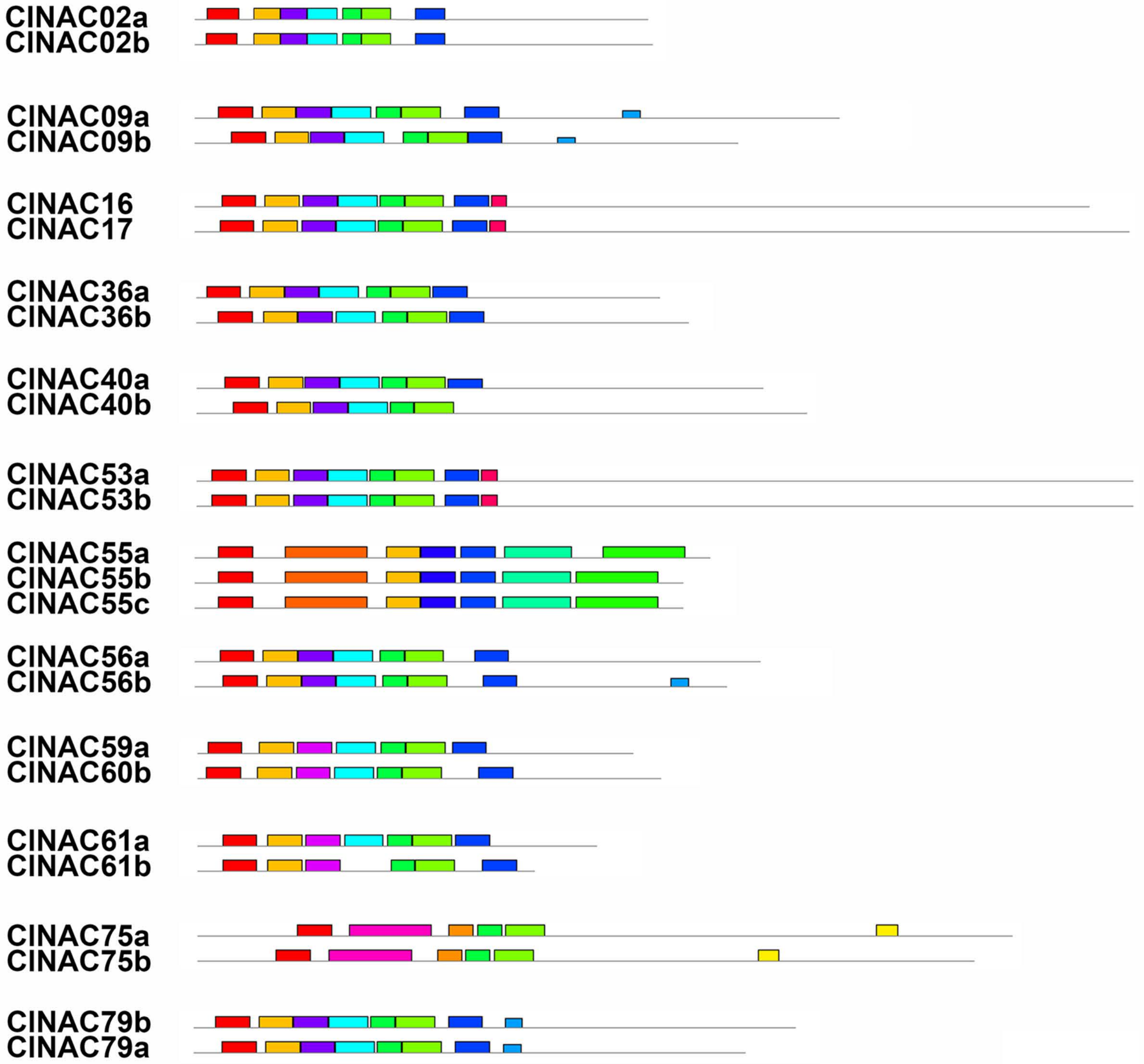
CINAC02a
CINAC02b
CINAC09a
CINAC09b
CINAC16
CINAC17
CINAC36a
CINAC36b
CINAC40a
CINAC40b
CINAC53a
CINAC53b
CINAC55a
CINAC55b
CINAC55c
CINAC56a
CINAC56b
CINAC59a
CINAC60b
CINAC61a
CINAC61b
CINAC75a
CINAC75b
CINAC79b
CINAC79a

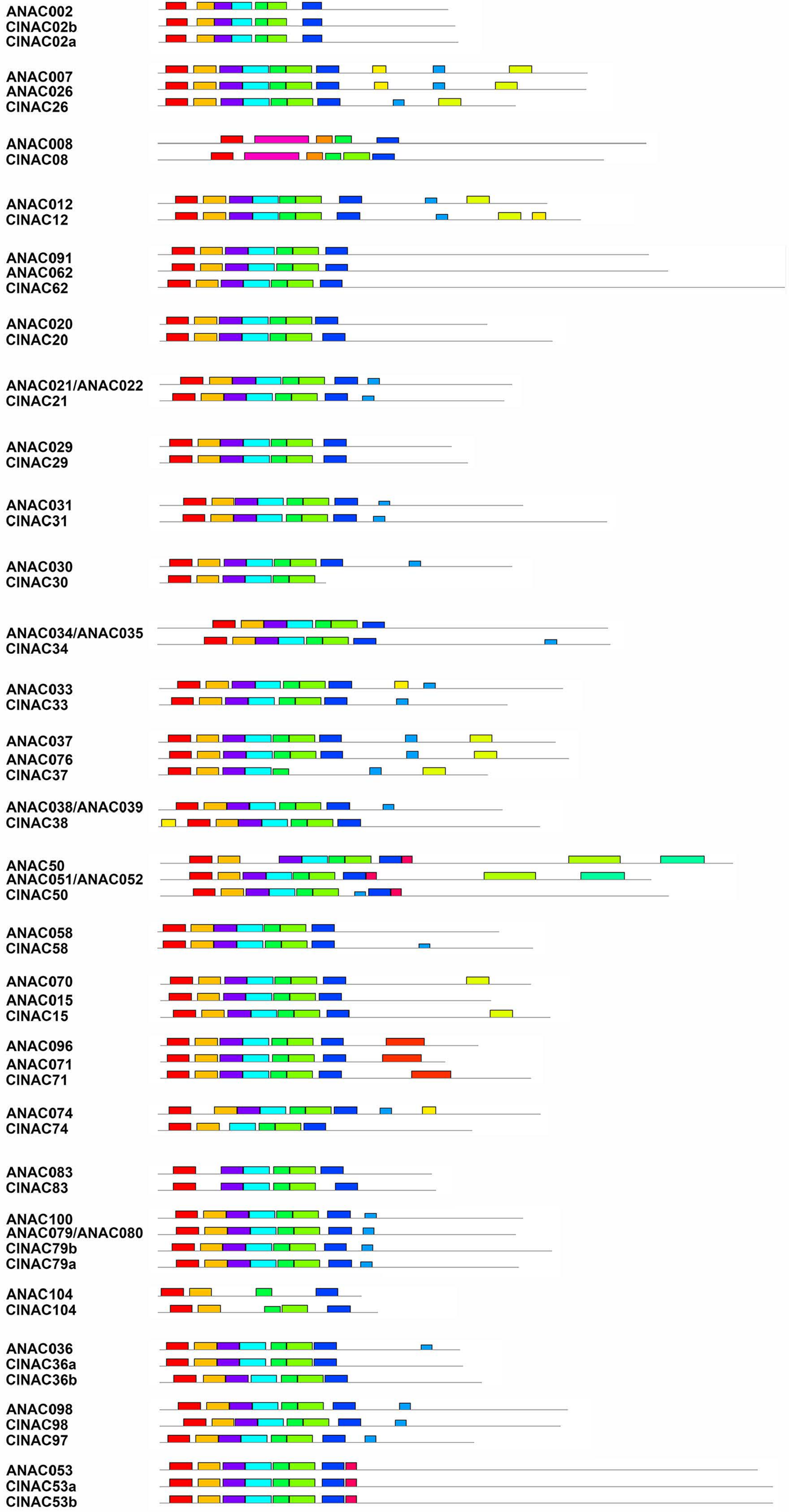
ANAC002
CINAC02b
CINAC02a
ANAC007
ANAC026
CINAC26
ANAC008
CINAC08
ANAC012
CINAC12
ANAC091
ANAC062
CINAC62
ANAC020
CINAC20
ANAC021/ANAC022
CINAC21
ANAC029
CINAC29
ANAC031
CINAC31
ANAC030
CINAC30
ANAC034/ANAC035
CINAC34
ANAC033
CINAC33
ANAC037
ANAC076
CINAC37
ANAC038/ANAC039
CINAC38
ANAC50
ANAC051/ANAC052
CINAC50
ANAC058
CINAC58
ANAC070
ANAC015
CINAC15
ANAC096
ANAC071
CINAC71
ANAC074
CINAC74
ANAC083
CINAC83
ANAC100
ANAC079/ANAC080
CINAC79b
CINAC79a
ANAC104
CINAC104
ANAC036
CINAC36a
CINAC36b
ANAC098
CINAC98
CINAC97
ANAC053
CINAC53a
CINAC53b

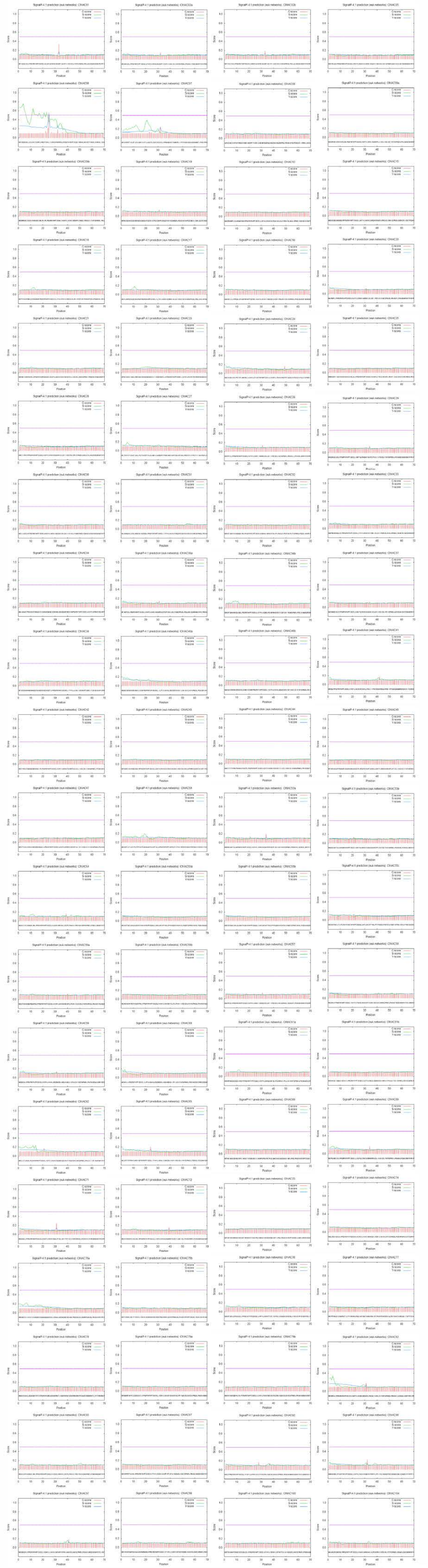

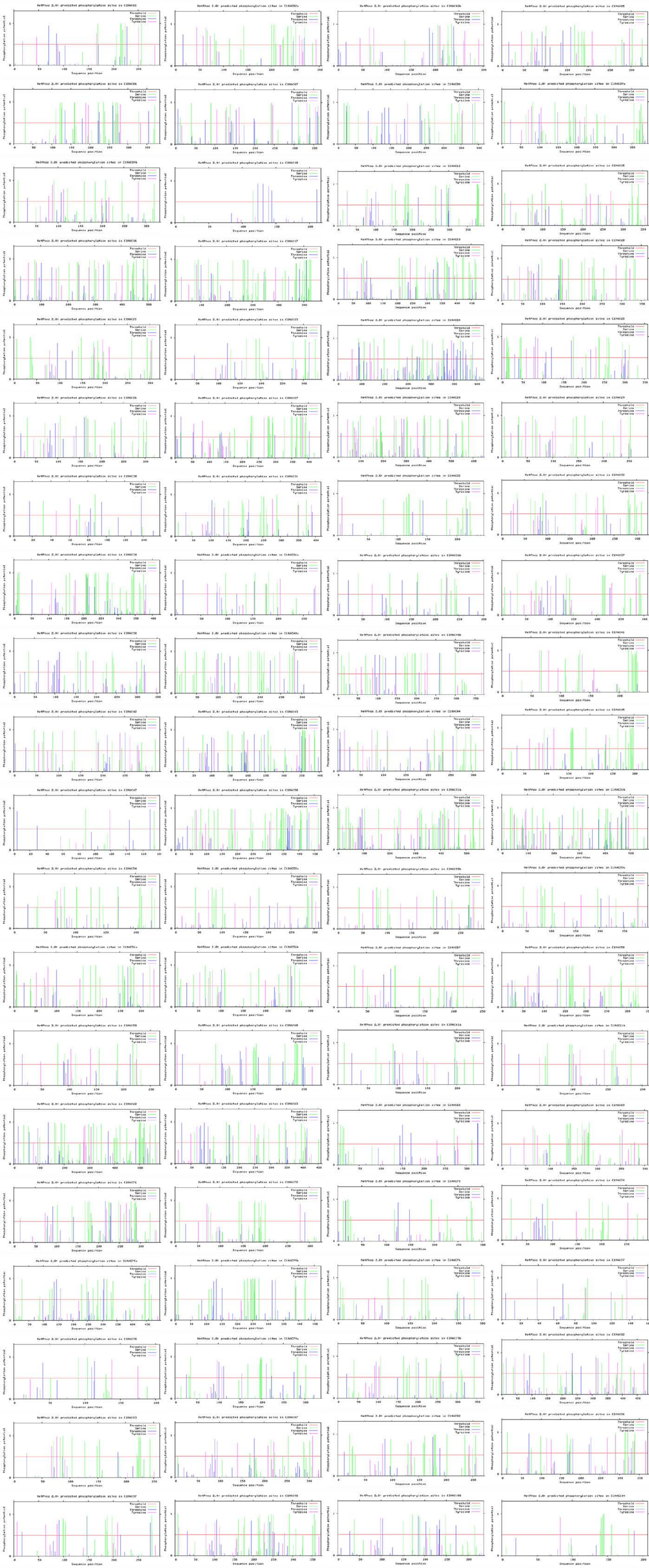
Phosphorylation potential
Sequence position
Threshold
Serine
Threonine
Tyrosine

Table S1. Sequence logos for the conserved motifs of watermelon NAC domain proteins.

Motif	E-value	Sites	Width	Logo
**Motif 1**	4.2e-906	80	22	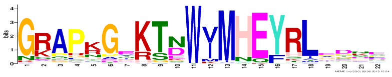
**Motif 2**	1.9e-1031	77	20	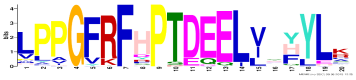
**Motif 3**	7.1e-876	60	21	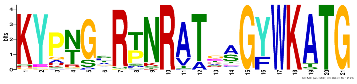
**Motif 4**	3.3e-619	65	21	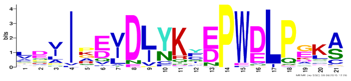
**Motif 5**	2.5e-482	74	15	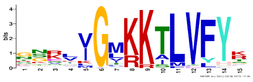
**Motif 6**	3.2e-403	62	15	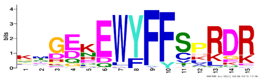
**Motif 7**	7.4e-368	71	21	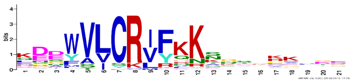
**Motif 8**	7.90E-110	7	34	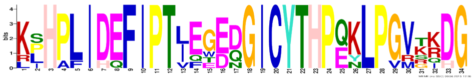
**Motif 9**	2.40E-82	4	50	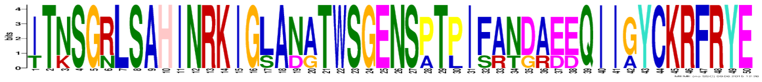
**Motif 10**	2.40E-49	3	50	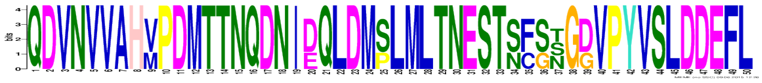
**Motif 11**	6.10E-49	7	21	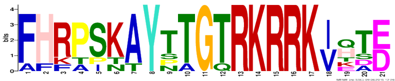
**Motif 12**	1.00E-43	3	50	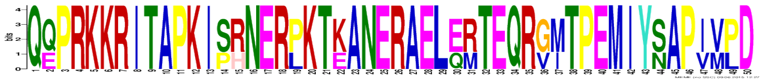
**Motif 13**	6.90E-42	4	29	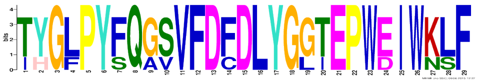
**Motif 14**	2.20E-38	7	15	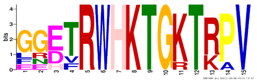
**Motif 15**	1.90E-26	5	15	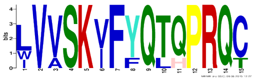
**Motif 16**	2.20E-24	47	6	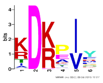
**Motif 17**	1.10E-20	7	16	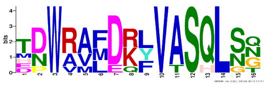
**Motif 18**	2.90E-20	5	14	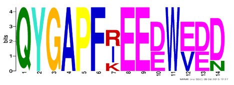
**Motif 19**	2.20E-13	47	8	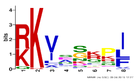
**Motif 20**	1.80E-08	5	8	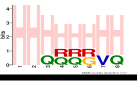

Table S3. Number of different cis-elements presented in the promoter regions of the CINAC genes.

Gene name	MBS	LTR	ABRE	TCA-element	HSE	ERE	ARE	TGACG-motif	CGTCA-motif
CINAC01	1		1		1	1		1	1
CINAC02a	1		4		1		1	4	4
CINAC02b	1		7	1	2	1	3	1	1
CINAC05	1		2	2	1	1			
CINAC06	1						2		
CINAC07	1		2	2	1	1			
CINAC08					1		3	1	1
CINAC09a			1	1	1	2			
CINAC09b					1				
CINAC10				1			1	1	1
CINAC12				3		1	1		
CINAC15			3		1		2	1	1
CINAC16		1				2		1	1
CINAC17	1					1	2		
CINAC18		1				1			
CINAC20				4	1		3	3	3
CINAC21	1		1	2				1	1
CINAC23			1		1		1		
CINAC24					1	1	1	2	2
CINAC25			1	2		3	3		
CINAC26	1				2	1	5		
CINAC27	1		2				3		
CINAC28		1		3					
CINAC29				2	3	1	2		
CINAC30	1	1	1	1	1		2		
CINAC31		1	1	2			2		
CINAC32			1	1		2		1	1
CINAC33				1	1		1		
CINAC34				4	1			1	1
CINAC36a	2	1			1	2	1		
CINAC36b	1			1		1	1		
CINAC37	2		2		1		1	1	
CINAC38		1			1	1	1		
CINAC40a			1				1	1	1
CINAC40b	2		2		1		1	1	1
CINAC41	1			1			1	2	2
CINAC42				1	2		2		
CINAC43		1		5			1	1	1
CINAC44		1		1	2			1	1
CINAC45				1	1		2		
CINAC47	2		1		1	1		1	1
CINAC50	1					1	1		
CINAC53a			2					2	2
CINAC53b			1				2	4	4
CINAC54					2				
CINAC55a							1		
CINAC55b		1		1	2		1	1	1
CINAC55c		1	1	1		1	1	2	2
CINAC56a			3		1	1		2	
CINAC56b	3		1		2			1	1
CINAC57				1					
CINAC58				1	1				
CINAC59					1		2		
CINAC60				1			1		
CINAC61a	1			1			1	1	
CINAC61b				1	1				
CINAC62				4	1		3	3	3
CINAC63				1			2	1	1
CINAC68		1		1	1		1	1	
CINAC69			8				2	2	
CINAC71					2	1	1		
CINAC72			3		1		3	2	2
CINAC73	1			1	2		3		
CINAC74	1		1	1			1	2	2
CINAC75a	1		2	3	1		1		
CINAC75b	1			1			3		
CINAC76		1	5	1	4			1	1
CINAC77	1	2		1		1	1		
CINAC78					2	1			
CINAC79a	1		2	1				1	1
CINAC79b				2	1		1	1	1
CINAC82	3			2					
CINAC83				1				1	1
CINAC87		1		1	1	2			
CINAC92	1			1	2		2	2	
CINAC96	2		1	1				3	3
CINAC97			1	1	2				
CINAC98					1		1	1	1
CINAC100					2			1	1
CINAC104				2			1	2	2

Table S4. Subcellular localization predictions for ClNACs

Protein name	Nucl	Cyto	Mito	Chlo	Vacu	Extr	Golg	Cysk	Plas	Pero
ClNAC01	5	5	2						2	
ClNAC02	4	6		1						2
ClNAC03	3									
ClNAC04	3	2	4	3	1					
ClNAC05	3	1		2						7
ClNAC06	4	3								6
ClNAC07	1	2	1	7		2				
ClNAC08	4	6							2.5	
ClNAC09	13									
ClNAC10	8	4		1						
ClNAC11	10	1							2	
ClNAC12	9	2		1	1					
ClNAC13	11	1			1					
ClNAC14	8		1	2			2			
ClNAC15	7	1		1	2					
ClNAC16	4	2		5					2	
ClNAC17	12			1						
ClNAC18	14									
ClNAC19	13									
ClNAC20	3		1		1		1.5		6.5	
ClNAC21	14									
ClNAC22	12.5									
ClNAC23		2	4	1		2		1		
ClNAC24	13									
ClNAC25	3	4						7		
ClNAC26	9	2		1		1				
ClNAC27	10	1		1		1				
ClNAC28	11			2						
ClNAC29	7	5						2		
ClNAC30	12	1								
ClNAC31	13									
ClNAC32	6	3	2							2
ClNAC33	9	1		1					1.5	
ClNAC34	6	5	1	1						
ClNAC35	7	4		1		1				
ClNAC36	10	3								
ClNAC37	7	4							2	
ClNAC38	14									
ClNAC39	8.5			2					1.5	
ClNAC40	3	1		8					1	
ClNAC41			2	5	3	1			1	
ClNAC42	6	4	1	2						
ClNAC43	7	5		1						
ClNAC44	2	10		2						
ClNAC45	13									
ClNAC46	14									
ClNAC47	3	3		3	1		2		1	
ClNAC48	12		1							
ClNAC49	12			1						
ClNAC50	3	1	1	3		1		1	3	
ClNAC51	11	1		1						
ClNAC52	13									
ClNAC53	13									
ClNAC54	12		1							
ClNAC55	13									
ClNAC56	12								1	
ClNAC57	9	2		2						
ClNAC58		13								
ClNAC59	1	11	1							
ClNAC60		2		9						2
ClNAC61	5	4	1		1				2	
ClNAC62	14									
ClNAC63	12		2							
ClNAC64	4	1		8						
ClNAC65	8	3	1	1						
ClNAC66	12			2						
ClNAC67	10	1	2							
ClNAC68	7	4						2		
ClNAC69	13									
ClNAC70	5	4	2	2						
ClNAC71	9			1	1	1			1	
ClNAC72	9			2		2				
ClNAC73	5	2		1		4				
ClNAC74	13									
ClNAC75	8	1	2	2						
ClNAC76	6	3	1	1						2
ClNAC77	11			1		1				
ClNAC78	10	1		2						
ClNAC79	6	1	4.5	1						
ClNAC80	3	6	2	2						

Nucl: nucleus; **Cyto**:cytoplasm; **Mito**: mitochondria; **Chlo**: chloroplast; **Plas**: plasma membrane; **extr**: extracellular; **Pero**: peroxisome; **Vacu**: vacuole; **Cysk**: cytoskeleton；**Golg**：golgiosome.

Table S5. Phosphorylation site predictions in 80 CINACs.

Protein name	Ser	Thr	Tyr
CINAC01	8	4	3
CINAC02	6	4	7
CINAC03	18	3	8
CINAC04	10	9	9
CINAC05	11	7	4
CINAC06	14	7	2
CINAC07	17	6	5
CINAC08	16	9	2
CINAC09	16	5	4
CINAC10	9	3	6
CINAC11	1	5	0
CINAC12	16	6	1
CINAC13	22	6	8
CINAC14	29	7	2
CINAC15	21	9	3
CINAC16	30	6	6
CINAC17	19	4	8
CINAC18	15	2	5
CINAC19	16	7	5
CINAC20	22	6	9
CINAC21	23	8	4
CINAC22	10	2	6
CINAC23	3	4	2
CINAC24	16	17	5
CINAC25	7	4	0
CINAC26	14	6	4
CINAC27	14	4	4
CINAC28	27	6	2
CINAC29	8	6	5
CINAC30	8	4	2
CINAC31	12	6	4
CINAC32	9	7	4
CINAC33	15	6	6
CINAC34	20	5	3
CINAC35	11	4	8
CINAC36	12	7	5
CINAC37	20	2	5
CINAC38	2	4	1
CINAC39	20	8	4
CINAC40	26	7	4
CINAC41	30	6	8
CINAC42	9	2	6
CINAC43	10	3	4
CINAC44	8	2	4
CINAC45	15	4	5
CINAC46	14	3	5
CINAC47	27	9	7
CINAC48	10	3	1
CINAC49	13	5	3
CINAC50	19	11	3
CINAC51	13	3	3
CINAC52	17	8	5
CINAC53	10	1	3
CINAC54	20	1	6
CINAC55	12	3	1
CINAC56	7	5	2
CINAC57	10	2	2
CINAC58	3	4	2
CINAC59	3	3	2
CINAC60	7	3	2
CINAC61	16	6	0
CINAC62	19	5	1
CINAC63	15	2	2
CINAC64	12	3	1
CINAC65	13	2	4
CINAC66	16	2	2
CINAC67	8	4	5
CINAC68	8	5	4
CINAC69	15	9	3
CINAC70	8	6	2
CINAC71	8	2	5
CINAC72	17	4	3
CINAC73	12	6	6
CINAC74	16	6	6
CINAC75	11	5	4
CINAC76	7	8	1
CINAC77	14	7	2
CINAC78	15	3	3
CINAC79	9	6	7
CINAC80	7	1	1

Table S6. Primer sequences of ClNAC genes for RT-PCR and qRT-PCR analyses.

Gene symbol	Primer	
	F	R
ClNAC01	AGAATGCACACCGACTCCTC	TTGGGTTGACTTTGGACCTC
ClNAC02a	AAGGGCGTAATCGAGAAACA	GAAACTGCCCTTTCGTTCAG
ClNAC02b	CAATACCGCGATTACACACG	TTCCAATTCCTTCCATTTCG
ClNAC05	AAATGCCACTCCACAGGAAG	GTTCCTCGTACCAACACGGT
ClNAC06	GAATCCTCCGATTCCACCTC	TGCAAATGACTGAGGCAGAC
ClNAC07	CTTTGCAAGCATGAGGTTGA	CATAGGTGCACGACAAGACG
ClNAC08	TTGATCCTGCAAACATCGAA	CCGAAATCAAGCTGGCTAAG
ClNAC09a	TGGATGATGCATGAATTTCG	TCTTTGAGCTGTGGAGTTGG
ClNAC09b	CGTGGACAGGAGAGAAGGAG	TGGGACGATCAGTTCCAGTA
ClNAC10	CAAAGATTCATCCACTCATCCA	TGTCGGATTTCACCATCTTTC
ClNAC12	GACGAAAGAGCCATGGAAGA	CTCATGCCCATATCCCAATC
ClNAC15	ATGTTGGATCGGCTCGTTAC	TCACTGATGACGACCCATGT
ClNAC16	AATGATGGAGGTGGTTCTGC	GATGCAGGAGTGGTCGGTAT
ClNAC17	ACTCCTTATCCCTGGGTGCT	GCTGCTGCTCGGAATTAGAC
ClNAC18	CAACTGGGAGGAGCTCTTTG	CCAGCTTAAACCCGTATCCA
ClNAC20	TTCACATTGATTGCCCTTCA	CGAGCTCTAATGGCGGATAG
ClNAC21	AGTACGCGACTGGGCTTAGA	ACGAGCTGATTGGCCTTATG
ClNAC23	GCCTCGAAAGCTTCATCATC	ATAGTTCGTGTCGGTCGCTT
ClNAC24	GCCTGAGATGAAGCAGGGTA	CGCCCATAACACATCATCAG
ClNAC25	CCACCGACGAGGAATTAGTG	GTCCCATGGATCGAACTTGT
ClNAC26	GAGCTCGATTCTCCAACCAC	AGTGCAACTCGAGCTCTTGC
ClNAC27	ATTAACCAGGACAGGGCACA	CTTGATGCACCACCTCCTTC
ClNAC28	TTTGATGTGGAAAGCTCGTG	TTCCATGCAATTCTTGTGGA
ClNAC29	GGCTTCAGATTTCACCCAAC	TTATGGAAACAGGACATGGCT
ClNAC30	AAATTTGGTTACGAGGAGCAAA	ACTGCCTTATCCCTCCCTGT
ClNAC31	GAGACCGGAAATACCCAACA	GGGATCCATTTGAAGCACTG
ClNAC32	TAAGCCAAGGGATCAACCAG	TGAGTTGGGAGTTGTTGCAG
ClNAC33	CGCAGCTGTTTAGTCCAGAG	CCGCAACTTAACCTCCAGAT
ClNAC34	GCTTCCATCCCACTGAAGAA	AAGCTCCCAAGGGTCATAGC
ClNAC36a	AAGAGGCTGCAGTTTGATCC	CTCTCCAATCCTTGCCAATC
ClNAC36b	GGCGAAGAATGGTGAGAGAG	CTTTCCAGTACCCAATTCCG
ClNAC37	AGCAAGAGATGGAACAAGCTG	TCTTCATTGCACTCGGACAC
ClNAC38	TTGGAATGAAGAAGACGTTGG	CACCCATTCGTCCTTAGCTG
ClNAC40a	AAGGCGGCTTCGAGTAAAGT	AATTTGCTCCAGCGAATGAG
ClNAC40b	TGCTTCGGATCCTTTACCAC	GCAGAACCTTGGTTCGGTAG
ClNAC41	CTGCCTTGCCTGCTTCTATC	CCTGCGCCTGTAGGAATATC
ClNAC42	CGTCTCCCTCCTCCTAATCC	TCTGCTTCTTGGGCAAAGTT
ClNAC43	AAGTACCCGACTGGAACTCG	CAAGCGTCTTTCTCATTCCA
ClNAC44	TCATTGCATTGGCCTAAAGA	GGCGAAGTGAGAAGTGGAAC
ClNAC45	ACAGTGTGAGGCCAAACAGA	GCCTTTACCAGCAGATCCAC
ClNAC47	AAAGTGAGCAATGTGGGAGA	TGTCAATGCCTGTTGCTTTC
ClNAC50	GACCTCCAAATGGACATCGT	CCGTCTGCAATCTCTTCTCC
ClNAC53a	CGCAGATGGTGATAATGCAG	CAGTGTGTTTGCTGGCAGAT
ClNAC53b	AAACAATCGTCCATGGCTTC	CTGGCACGTTTCAAGAATGA
ClNAC54	GGCTCAAGAACTGATTGGGT	CCGTCATTAAAGGTGGAAGG
ClNAC55a	CGTCAAATTTAGGCCTACCG	GCTCGGTTCCACCATAAAGA
ClNAC55b	CGTCAAATTTAGGCCTACCG	GCTCGGTTCCACCATAAAGA
ClNAC55c	TGCCATTGAACCATTACGTG	GCTCGGTTCCACCATAAAGA
ClNAC56a	ATGGAGAGCACCGACTCGT	GAACCACGAGCTCTTCATCG
ClNAC56b	CTTCCAGCTAAGGCGAGCTA	TTCCAGTCGCCTTCCAATAC
ClNAC57	GATGGCAGCTTCATCTTCTTG	GCTGATGAATTGGAAGCACA
ClNAC58	CGATGGGAGAGAAAGAATGG	GACCAGTGCCAAAGATTTCC
ClNAC59	GCGTGCAAGAGAGATGAAGA	CCAGTTGCTTTCCAATAGCC
ClNAC60	CTCAGTTCGCTGCTGGAAG	GAGCGATTGAGGTTGGGTTA
ClNAC61a	AACCAAATGGAAGATGAACGA	AAAGCATCCGGAAATCACAT
ClNAC61b	GAGCCGTTCAATCTCTCCAG	CCTTCCAATACCCAGATCCA
ClNAC62	ATGCAGAGTCCAGGAGCAGT	AAGGATTGAAGGTGGCAGTG
ClNAC63	GAACCAACAAAGTGATCGCA	TCTGGGTGAAGGTGATACTCG
ClNAC68	ATCCTGTGGCCATCAGATTC	ATTTCACTCCAGCTGGCAAC
ClNAC69	GACGATTTCTTCGCGTATCC	GTTCCGGCACTGAGTAGAGC
ClNAC71	TCGTCTCAGACCAATTTCCTG	CGTGCTATACCGAGACATGG
ClNAC72	GCAACCACTGAGGAACACG	GGAGTTGGAGAAACTTTGCG
ClNAC73	AGCCAGAGAAGACAAACTGGA	TGGAGTGAGTTCCCACATTG
ClNAC74	ACTTCGAGCGTGTTCCATCT	TTGTAGTCACAAGGCATCGC
ClNAC75a	AATTGGTCGGAGAGAAGCCT	TCTCCCGTCGACTCAAATTC
ClNAC75b	CTCCTCTCCACCATTCCTCA	AAGCAGACCTCCCTCCTAGC
ClNAC76	AGGGAGAACAGAGGACGGAT	CTCCTCTTCATCCCAAGCAC
ClNAC77	TCACACTTTCCGTTTGATGC	CAGCCCATTTGAGTCGTTG
ClNAC78	TGGCAAAGCTAAATGGGAAG	TCCAGTTGCTTTCCAATATCC
ClNAC79a	GCCATTGAAGGATTCCTCTTC	ATCCATGGTGTTGGAGAAGC
ClNAC79b	ATCGCCATGGATTCTCAAAG	TTGGCGGTGAATACATTGAA
ClNAC82	TTTGGAAGATGCAGCTCAGA	TCAACCTCCTCCAGACCATC
ClNAC83	TTTCTTCAGCACGAGAGAAGC	TCTTCAACCCAACCACTTGA
ClNAC87	GGAAGCAACACTTCCATCGT	TTATCGACGTAGTCCCACCAG
ClNAC92	AGAGCAACAGTCGCTTCTCAG	GTTGTTGACATCGGTGGTGA
ClNAC96	AGTTGGATGGAGTCGGAGTG	TGCTGCTTCCAATGCTACAG
ClNAC97	AACCGGGTTGAGAACGAAC	TTGGAGCTCTGCCTTTGTAGA
ClNAC98	AGAGGAAGAGCACCCAAAGG	CCCTTGAAATCACCCACTCA
ClNAC100	TGGAAGACGCAGAATGAGTG	CGATGGCAGTGAAATTGGTA
ClNAC104	CGCCTCTTCTTCTTCCTCCT	TCATCCAAACACGACAGCTC
